# Subclinical carotid artery atherosclerosis and cognitive function in older adults

**DOI:** 10.1186/s13195-022-00997-7

**Published:** 2022-05-07

**Authors:** Felice Lin, Judy Pa, Roksana Karim, Howard N. Hodis, S. Duke Han, Victor W. Henderson, Jan A. St. John, Wendy J. Mack

**Affiliations:** 1 Department of Population and Public Health Sciences, Keck School of Medicine, University of Southern California, Soto Street Building Suite 202Y, 2001 North Soto St, Los Angeles, CA 90089 USA; 2 Mark and Mary Stevens Neuroimaging and Informatics Institute, University of Southern California, Los Angeles, CA USA; 3 Atherosclerosis Research Unit, Keck School of Medicine, University of Southern California, Los Angeles, CA USA; 4 Department of Medicine, Keck School of Medicine, University of Southern California, Los Angeles, CA USA; 5 Department of Family Medicine, University of Southern California, Los Angeles, CA USA; 6 Department of Neurology, University of Southern California, Los Angeles, CA USA; 7 Department of Psychology, University of Southern California, Los Angeles, CA USA; 8 School of Gerontology, University of Southern California, Los Angeles, CA USA; 9 Departments of Epidemiology and Population Health and of Neurology and Neurological Sciences, Stanford University, Stanford, CA USA

**Keywords:** Cognitive function, Subclinical atherosclerosis, Carotid artery intima-media thickness, Middle- to older-aged adults

## Abstract

**Background:**

The combined effects of increased life expectancy and the considerable number of persons reaching old age will magnify the dementia epidemic in the USA. Demonstration that subclinical atherosclerosis precedes and is associated with cognitive impairment suggests a modifiable risk factor for age-associated cognitive impairment and dementia. The purpose of this study is to determine whether subclinical atherosclerosis as measured by carotid artery intima-media thickness (CIMT) is associated with changes in cognitive function over time in older adults.

**Methods:**

This study combined longitudinal data from three clinical trials conducted between 2000 and 2013: the B-Vitamin Atherosclerosis Intervention Trial (BVAIT), the Women’s Isoflavone Soy Health (WISH) trial, and the Early versus Late Intervention Trial with Estradiol (ELITE). Participants were recruited from the general population in the Greater Los Angeles area and were free of cardiovascular disease and diabetes; no cognitive or psychiatric exclusion criteria were specified. The same standardized protocol for ultrasound image acquisition and measurement of CIMT was used in all trials. CIMT measurements performed at baseline and 2.5 years were used in these analyses. Cognitive function was assessed at baseline and 2.5 years using a battery of 14 standardized cognitive tests. All clinical trials were conducted at the University of Southern California Atherosclerosis Research Unit, Los Angeles, and had at least 2.5 years of cognitive follow-up.

**Results:**

A total of 308 men and 1187 women, mean age of 61 years, were included in the combined longitudinal dataset for the primary analysis. No associations were found between CIMT and cognitive function at baseline or at 2.5 years. There was a weak inverse association between CIMT measured at baseline and change in global cognition assessed over 2.5 years (*β* (SE) = − 0.056 (0.028) units per 0.1 mm CIMT, 95% CI − 0.110, − 0.001, *p* = 0.046). No associations between CIMT at baseline and changes in executive function, verbal memory, or visual memory were found.

**Conclusions:**

In this sample of healthy older adults, our findings suggest an association between subclinical atherosclerosis and change in global cognitive function over 2.5 years. Stronger associations were observed longitudinally over 2.5 years than cross-sectionally. When analysis was stratified by age group (<65 and ≥65 years old), the inverse association remained statistically significant for participants in the older age group. Subclinical atherosclerosis of the carotid artery may be a modifiable correlate of cognitive decline in middle and older age.

**Trial registration:**

BVAIT, NCT00114400. WISH, NCT00118846. ELITE, NCT00114517.

## Background

Alzheimer’s disease and related dementias are major public health concerns and the recognition of modifiable risk factors has revealed new avenues for dementia risk reduction. Indeed, evidence of a decline in the prevalence of dementia in the USA may be attributed in part to an increased awareness about controlling cardiovascular risk factors, particularly in middle-aged adults [1].

Previous studies have shown that carotid artery atherosclerosis is a modifiable risk factor for cognitive impairment [2]. Greater amounts of carotid arterial plaque have been linked to an increased risk of developing dementia [3]. Even mild carotid artery atherosclerosis, or subclinical atherosclerosis, has been shown to be associated with poorer cognitive function in middle-aged adults after adjusting for other vascular risk factors [4, 5].

Carotid artery intima-media thickness (CIMT), a measurement of the thickness of the two inner layers of the carotid artery, is a commonly used and validated research measure of atherosclerosis in the subclinical stages [6]. Past research has shown that thicker CIMT is associated with poorer cognitive performance, generally, and in certain cognitive domains. However, with regard to global cognition, the findings have been inconsistent. For example, while a few studies have not found an association between thicker CIMT and global cognition [5, 7, 8], one study found an inverse association [9]. In addition, past research has found that greater CIMT is longitudinally associated with cognitive decline in adults aged 65 years and older; results have been mixed for middle-aged adults [10].

There are several possible reasons for the lack of consistency in the findings. One is that the various instruments used to evaluate the same cognitive domain differ in their sensitivity to the assessment of cognitive decline. In addition, some studies used only a single instrument while others employed multi-test batteries to assess cognitive function. Differences in study populations and the image acquisition and measurement methods for subclinical atherosclerosis may also contribute to the lack of consistency in the findings [8, 11, 12]. Finally, most previous studies have only examined the cross-sectional associations between CIMT and cognitive function. Few studies have been longitudinal in nature.

To evaluate the association between subclinical atherosclerosis and cognitive function in older age, this current study had a larger study population using combined data from three clinical trials and examined cognitive change over time. Participants in each of these clinical trials completed the same extensive battery of standardized neuropsychological tests at baseline and at follow-up visits, allowing for the examination of longitudinal associations. All trials used a well-characterized measure of subclinical atherosclerosis, CIMT of the right common carotid artery; ultrasound imaging and CIMT measurement were completed using the same standardized protocol in all three trials. Additionally, we controlled for a number of potential confounding effects of cardiovascular, genetic, and demographic risk factors. We hypothesized that thicker CIMT measured at baseline is associated with greater declines in cognitive function over an average of 2.5 years among older adults.

## Methods

### Study design

This longitudinal analysis used combined data from three clinical trials: B-Vitamin Atherosclerosis Intervention Trial (BVAIT, NCT00114400), Women’s Isoflavone Soy Health (WISH, NCT00118846) trial, and Early versus Late Intervention Trial with Estradiol (ELITE, NCT00114517). Participants in all three trials were of similar cardiovascular health and did not have diabetes or clinical signs/symptoms of cardiovascular disease. Cognitive function was measured by one trained psychometrist using the same battery of 14 neuropsychological tests at baseline and 2.5 years in all three trials.

#### BVAIT design

BVAIT was a randomized, double-blinded, placebo-controlled trial conducted from November 2000 to June 2006 [13]. The trial was designed to examine whether B-vitamin supplementation would reduce the progression of early subclinical atherosclerosis in individuals over 40 years of age with higher levels of total homocysteine. Eligible men and women had fasting total homocysteine ≥ 8.5 μmol/L. A total of 506 participants were randomized to daily high-dose vitamin B supplementation (folic acid 5 mg + vitamin B_12_ 0.4 mg + vitamin B_6_ 50 mg) or matching placebo in a 1:1 ratio within two strata of baseline CIMT (< 0.75 mm, ≥ 0.75 mm).

#### WISH design

WISH was a randomized, double-blinded, placebo-controlled, randomized trial conducted from April 2004 to March 2009 [14]. WISH was designed to determine whether isoflavone-rich soy protein supplementation had an effect on the progression of subclinical atherosclerosis in healthy postmenopausal women. A total of 350 women were randomized to treatment (25 g soy protein containing 91 mg aglycone equivalents of naturally occurring isoflavones and respective glycosides [154 mg total isoflavone conjugates plus aglycone equivalents] of genistein, daidzein, and glycitein) or matching placebo in a 1:1 ratio within two strata of baseline CIMT (< 0.75 mm, ≥ 0.75 mm).

#### ELITE design

ELITE was a randomized, double-blinded, placebo-controlled trial conducted from July 2005 to February 2013 [15]. ELITE was designed to examine the effects of oral 17β-estradiol on the progression of subclinical atherosclerosis and cognitive decline in healthy postmenopausal women. A total of 643 women were randomized to treatment (oral 17β-estradiol 1 mg/day plus micronized progesterone [45 mg] as a 4% vaginal gel for women with a uterus) or placebos in a 1:1 allocation ratio within strata defined by the length of time since menopause (early postmenopause, < 6 years past menopause; late postmenopause, ≥ 10 years past menopause).

Participants for these clinical trials were recruited from the general population in the Greater Los Angeles area mainly through media advertisements. All studies had CIMT measurements performed at baseline and every 6 months after and had at least 3 years of follow-up. No cognitive or psychiatric exclusion criteria were specified.

**Fig. 1:**
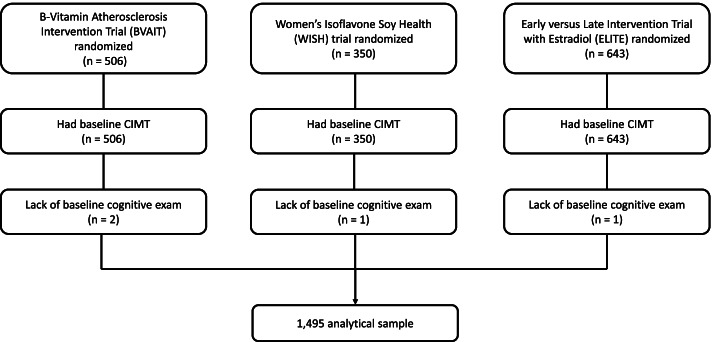
Clinical trial randomization, baseline CIMT measurements and cognitive exams and cognitive exams

The current study pooled data from 1495 men and women after 4 participants were excluded due to missing baseline cognitive data (Fig. 1).

### CIMT assessment

In each of the trials, high-resolution B-mode ultrasound images of the right distal common carotid artery were obtained as previously described at baseline and every 6 months during follow-up [16]. From the images, CIMT was measured using software that was developed at the University of Southern California (USC) Atherosclerosis Research Unit (ARU) for longitudinal measurements of atherosclerosis changes [17, 18]. At each visit, CIMT was calculated as the average of multiple individual measurements between the intima-lumen and the media-adventitia interfaces along a 1-cm length of the right common carotid artery far wall [5]. This method standardized the location and distance over which CIMT was measured and ensured that the same measurements were obtained for each participant in all trials [18].

### Cognitive function assessment

The same battery of cognitive and neuropsychological assessments was administered by a single trained psychometrist in a standardized order to participants. The battery included the following 14 assessments [5, 19]:

- Symbol Digit Modalities Test
- Trail Making Test, Part B
- Judgment of Line Orientation, Form H
- Block Design, Wechsler Adult Intelligence Scale, 3rd Edition
- Letter-Number Sequencing, Wechsler Memory Scale, 3rd Edition
- Category Fluency (animal naming, 60 s)
- Boston Naming Test, 30-item version
- Shipley Institute of Living Scale, Abstraction Scale
- California Verbal Learning Test, 2nd Edition, 3-trial immediate recall and delayed recall
- Logical Memory, immediate recall and delayed recall
- Faces I (immediate recall) and Faces II (delayed recall), Wechsler Memory Scale, 3rd Edition

The assessments were selected for sensitivity to age-related changes in cognitive function and representation of different cognitive functions and abilities with a focus on executive function and verbal and visual memory [19]. For each participant in each trial, all test scores were converted to a standardized *Z* score at baseline and 2.5 years. The *Z* scores were calculated using the trial-specific baseline mean and standard deviation of each test. To calculate the composite scores, the *Z* scores for the appropriate cognitive test were summed and then weighted by the inter-test correlation matrix [5, 14, 15, 20]. The results from the 14 assessments were used to generate composite scores for executive function, verbal memory, visual memory, and global cognition. The executive function composite score was calculated as a weighted average of the following cognitive tests: Symbol Digit Modalities Test, Trail Making Test, Part B, Shipley Institute of Living Scale, Abstraction Scale, Letter-Number Sequencing, and Category fluency (animal naming, 60 s) tests. The verbal memory composite score was calculated as a weighted average of the California Verbal Learning Test, 2nd Edition (immediate and delayed recall) and Logical Memory (immediate and delayed recall) test. The visual memory composite score was calculated as a weighted average of the Faces I (immediate recall) and Faces II (delayed recall) tests. The Global Cognition Composite Score was calculated as a weighted average of all 14 assessments.

### Covariates

At the baseline visit for each clinical trial, demographic information (age, sex [male, female], race/ethnicity [non-Hispanic white, non-Hispanic black, Hispanic, Asian or Pacific Islander, other], education level [number of years], income level [annual income] and marital status [single, married, separated, divorced, widowed]), laboratory values (high-density lipoprotein (HDL) and low-density lipoprotein (LDL) cholesterol, glucose), lifestyle factors (current smoking status [yes, no], alcohol consumption [yes, no], and physical activity summary measures [weekly moderate activity hours, weekly vigorous activity hours]), use of anti-hypertensive and lipid-lowering medications [yes, no], body mass index (BMI), and blood pressure were collected. Three isoforms (ε2, ε3, and ε4) of the apolipoprotein E (ApoE) gene determined according to two nonsynonymous single nucleotide polymorphisms (rs429358 and rs7412) [21] were also assessed (TaqMan Assay-on-Demand Genotyping Service; Applied Biosystems). In addition, participants completed the Center for Epidemiological Studies Depression (CES-D) scale [22].

### Statistical methods

Because some CIMT and cognitive data were not captured exactly at the 2.5-year visit (primarily due to missed study visits), follow-up CIMT and cognitive measures were defined over a range of visits for the time point; the 2.5-year variable encompassed visit months 24–36.

#### Cross-sectional associations between CIMT and cognition at baseline and 2.5 years

Multivariable linear regression models were used to examine the cross-sectional association at baseline and at 2.5 years between CIMT and cognitive function domains. Covariates that were evaluated as potential confounders included age, sex, race, education, income, marital status, ApoE4 genotype, current smoking status, CES-D score, systolic blood pressure (SBP), and HDL and LDL cholesterol. Covariates were considered confounders if their inclusion in the models resulted in a ≥ 10% change in the CIMT regression coefficient. In models where height or weight was found to be a confounder, they were replaced with BMI as it incorporates both height and weight and is a better indicator of body composition [23]. Due to associations of vascular factors with atherosclerosis and cognition [24–26], vascular risk factors (BMI, SBP, HDL, LDL, and current smoking status) were included as relevant confounders in all models, regardless of whether or not they met the chage-in-estimate criterion. Indicator variables for study were also included in all models to account for differences between clinical trials. To assess potential trial treatment effects, a variable for trial treatment assignment (placebo, active B-vitamin [BVAIT], active isoflavone-rich soy protein [WISH], or active hormone treatment [ELITE]) was used. Product terms were used to test for interactions between CIMT and ApoE4 genotype as well as between CIMT and sex, and between CIMT and age group (participants < 65 years old and participants ≥ 65 years old). CIMT was modeled as a continuous variable. Since BMI, SBP, and HDL and LDL cholesterol are potential determinants of CIMT in the cognitive function pathway, the association was also examined without adjustment for these variables [27]. Associations with CIMT were reported per 0.1 mm, the approximate standard deviation from the study population [13, 28, 29].

#### Longitudinal associations between CIMT and cognition

A change variable was created for each dependent cognitive variable as the difference between the 2.5 year and baseline scores. Associations between baseline CIMT and the cognitive change variables were tested in models adjusting only for baseline cognitive scores and in multivariable linear regression models. Potential confounders were re-evaluated in the final models. Modeling procedures were completed as described above with the change in cognitive measure as the dependent variable. To examine the CIMT-cognition association by age, analyses of associations between baseline CIMT and the cognitive change variables were also stratified by age group (< 65 and ≥ 65 years old). The associations between change in CIMT and the cognitive change variables were also assessed.

## Results

Study participant characteristics at baseline are summarized in Table 1. Since ELITE and WISH enrolled only women, most (79%) study participants were women. The majority were non-Hispanic White (66%) and married (59%). Study participants had an average age of 61 years, were highly educated (mean 16 years of education), had an average annual income of $66,700, and were overweight (mean BMI 27 kg/m^2^). Over a quarter of participants (27%) carried the ApoE4 genotype. The average CES-D score at baseline was well below the cutoff score of 16 indicating concern for clinical depression, and only 13% scored above the cutoff [30]. Due to similar exclusion criteria for cardiovascular risk factors, all participants were of comparable cardiovascular health. The average CIMT at baseline was highest in the WISH trial but was otherwise similar across all three clinical trials (overall mean = 0.77 ± 0.12 mm).

**Table 1:** Baseline characteristics for study participants by clinical trial (*n* = 1495)

Variable	BVAIT (***n*** = 504)^**a**^	WISH (***n*** = 349)^**a**^	ELITE (***n*** = 642)^**a**^	Combined
	Mean ± SD or number (%)			
Age (years)	61.5 ± 9.9	61.4 ± 7.1	60.6 ± 6.9	61.1 ± 8.1
Sex				
Male	308 (61.1%)	0 (0%)	0 (0%)	308 (20.6%)
Female	196 (38.9%)	349 (100%)	642 (100%)	1187 (79.4%)
Race				
Non-Hispanic White	326 (64.7%)	222 (63.6%)	439 (68.4%)	987 (66.0%)
Non-Hispanic Black	75 (14.9%)	21 (6.0%)	60 (9.3%)	156 (10.4%)
Hispanic	55 (10.9%)	55 (15.8%)	90 (14.0%)	200 (13.4%)
Asian or Pacific Islander	45 (8.9%)	38 (10.9%)	53 (8.3%)	136 (9.1%)
Others	3 (0.6%)	13 (3.7%)	0 (0%)	16 (1.1%)
Education (# of years)	15.7 ± 2.0^b^	15.8 ± 2.0	16.0 ± 1.9	15.9 ± 2.0
Annual income	$64.6k ± $29.7k^b^	$65.3k ± $31.3k^b^	$69.0k ± $31.0k^b^	$66.7k ± $30.7k
Marital status				
Single, never married	45 (8.9%)^b^	33 (9.5%)	50 (7.8%)	128 (8.6%)
Married	323 (64.1%)^b^	197 (56.4%)	368 (57.3%)	888 (59.4%)
Separated	5 (1.0%)^b^	7 (2.0%)	12 (1.9%)	24 (1.6%)
Divorced	89 (17.7%)^b^	80 (22.9%)	171 (26.6%)	340 (22.8%)
Widowed	41 (8.1%)^b^	32 (9.2%)	41 (6.4%)	114 (7.6%)
Current smoker	17 (3.4%)^b^	8 (2.3%)	22 (3.4%)	47 (3.2%)
Alcohol user	221 (43.8%)	152 (43.6%)	313 (48.8%)	686 (45.9%)
Use of anti-hypertensives at baseline	190 (37.7%)	117 (33.5%)	220 (34.3%)	527 (35.3%)
Use of cholesterol lowering medication at baseline	81 (16.1%)	72 (20.6%)	126 (19.6%)	279 (18.7%)
BMI (kg/m^2^)	28.1 ± 4.9	26.6 ± 5.2	27.2 ± 5.4^b^	27.4 ± 5.2
Blood pressure (mmHg)				
Systolic	127.0 ±15.6	117.9 ± 13.9	116.5 ± 13.7^b^	120.4 ± 15.2
Diastolic	78.7 ± 8.9	75.0 ± 8.6	74.5 ± 8.4^b^	76.0 ± 8.8
ApoE4+	103 (22.2%)^b^	86 (25.7%)^b^	197 (30.9%)^b^	386 (26.9%)
CES-D score^c^	6.2 ± 6.6	7.3 ± 6.8^2^	8.3 ± 8.6	7.4 ± 7.6
CES-D score ≥ 16^d^	44 (8.7%)	40 (11.5%)	113 (17.6%)	197 (13.2%)
Average CIMT (mm, over 1 cm segment)	0.75 ± 0.15	0.81 ± 0.10	0.77 ± 0.11	0.77 ± 0.12
Executive function composite score	− 0.012 ± 1.369	− 0.005 ± 1.323	− 0.002 ± 1.360	− 0.006 ± 1.353
Verbal memory composite score	− 0.005 ± 1.317	− 0.006 ± 1.357	0.001 ± 1.348	− 0.003 ± 1.339
Visual memory composite score	0.001 ± 1.108	0.001 ±1.107	0 ± 1.103	0.001 ± 1.105
Global cognition composite score	0.014 ± 1.745	0.002 ± 1.715	− 0.001 ± 1.827	0.005 ± 1.773

^a^Two participants in BVAIT and 1 participant in both WISH and ELITE each did not complete the baseline cognitive exam

^b^Differing sample sizes: Education, BVAIT: *n* = 503. Income, BVAIT: *n* = 472; WISH: *n* = 319; ELITE: *n* = 597. Marital status, BVAIT: *n* = 503. Current smoker, BVAIT: *n* = 503. BMI, ELITE: *n* = 641. Blood pressure, ELITE: *n* = 640. ApoE4 genotype, BVAIT: *n* = 463; WISH: *n* = 334; ELITE: *n* = 637. CES-D score, WISH: *n* = 348

^c^CES-D scores range from 0-60, with higher scores indicating more depressive symptomatology [22﻿]

^d^A score of 16 or higher on the CES-D indicates concern for clinical depression [30﻿]

### Cross-sectional associations between CIMT and cognition at baseline and 2.5 years

CIMT was not associated with any of the cognitive function composite scores after adjustment for various confounders either at baseline or at 2.5 years (Table 2).

**Table 2:** Cross-sectional associations between CIMT and cognitive function at baseline and 2.5 years from multivariable linear regression models

Cognitive domain	Baseline		2.5 years		Model covariates
	***β*** (95% CI)^**a**^	***p***-value	***β*** (95% CI)^**a**^	***p***-value	
Executive function	0.0221 (− 0.0331, 0.0773)	0.43	− 0.0340 (− 0.1062, 0.0382)	0.36	Age, sex, race, education, income, marital status, ApoE genotype, BMI, SBP, HDL, LDL, current smoking status, study
Verbal memory	0.0146 (− 0.0457, 0.0749)	0.64	− 0.0488 (− 0.1259, 0.0292)	0.22	Age, sex, race, education, income, ApoE genotype, CES-D score, BMI, SBP, HDL, LDL, current smoking status, study
Visual memory	0.0462 (− 0.0059, 0.0983)	0.08	0.0158 (− 0.0479, 0.0796)	0.63	Age, sex, race, education, income, marital status, BMI, SBP, HDL, LDL, current smoking status, study
Global cognition	0.0440 (− 0.0303, − 0.1183)	0.25	− 0.0338 (− 0.1292, 0.0616)	0.49	Age, sex, race, education, CES-D score, BMI, SBP, HDL, LDL, current smoking status, study

^a^Beta estimates are reported in units per 0.1 mm of CIMT

In models without BMI, SBP, HDL, or LDL, CIMT was also not associated with any of the cognitive function composite scores. No significant interaction by ApoE4 genotype was found between CIMT associations with cognition (all interaction *p*-values ≥ 0.19). Similarly, no significant interaction by age group was found (all interaction *p*-values ≥ 0.30). There was a significant interaction by sex for the association between CIMT and verbal memory composite score at baseline (*p* = 0.018). When the analysis was stratified by sex, the CIMT association was in opposite directions but was not statistically significantly associated with verbal memory composite score at baseline among men (*β* = − 0.071 units per 0.1 mm CIMT, 95% CI − 0.156, 0.015, *p* = 0.10) or among women (*β* = 0.073 units per 0.1 mm CIMT, 95% CI − 0.009, 0.154, *p* = 0.08). Trial treatment assignment did not affect estimates of the cross-sectional associations between CIMT and cognitive function at 2.5 years.

### Longitudinal associations between CIMT and cognition

Higher CIMT at baseline was weakly associated with a decrease in global cognition composite score assessed over 2.5 years (*β* = − 0.056 units per 0.1 mm CIMT, 95% CI − 0.110, − 0.001, *p* = 0.046) after adjusting for baseline global cognition composite score, age, sex, race, education, baseline CES-D, baseline BMI, baseline SBP, baseline HDL cholesterol, baseline LDL cholesterol, current smoking status, and indicator variables for clinical trial. There were no significant associations between CIMT at baseline and other cognitive domains assessed over 2.5 years (Table 3). There were no significant interactions by ApoE4 genotype, age group, or sex in the longitudinal models (all *p*-values ≥ 0.21).

**Table 3:** Associations between baseline CIMT and change in cognitive function assessed over 2.5 years from multivariable linear regression models

Cognitive domain	***β*** (95% CI)^**a**^	***p***-value	Model covariates
Executive function	− 0.0179 (− 0.0548, 0.0190)	0.34	Baseline executive function composite score, age, sex, race, education, income, marital status, ApoE4 genotype, baseline BMI, baseline SBP, baseline HDL, baseline LDL, current smoking status, study
< 65 years old	− 0.0199 (− 0.0638, 0.0241)	0.37	
≥ 65 years old	− 0.0129 (− 0.0842, 0.0584)	0.72	
Verbal memory	− 0.0236 (− 0.0793, 0.0321)	0.41	Baseline verbal memory composite score, age, sex, race, education, income, ApoE4 genotype, baseline CES-D score, baseline BMI, baseline SBP, baseline HDL, baseline LDL, current smoking status, study
< 65 years old	− 0.0144 (− 0.0800, 0.0513)	0.67	
≥ 65 years old	− 0.0491 (− 0.1572, 0.0590)	0.37	
Visual memory	− 0.0231 (− 0.0640, 0.0177)	0.27	Baseline visual memory composite score, age, sex, race, education, income, marital status, baseline BMI, baseline SBP, baseline HDL, baseline LDL, current smoking status, study
< 65 years old	− 0.0356 (− 0.0831, 0.0119)	0.14	
≥ 65 years old	− 0.0160 (− 0.0983, 0.0664)	0.70	
Global cognition	− 0.0557 (− 0.1103, − 0.0100)	0.046	Baseline global cognition composite score, age, sex, race, education, baseline CES-D score, baseline BMI, baseline SBP, baseline HDL, baseline LDL, current smoking status, study
< 65 years old	− 0.0351 (− 0.1005, 0.0303)	0.29	
≥ 65 years old	− 0.1078 (− 0.2100, − 0.0056)	0.039	

^a^Beta estimates are reported in units per 0.1 mm of CIMT

In the analysis stratified by age group, the weak inverse association between baseline CIMT and change in global cognition composite score assessed over 2.5 years remained statistically significant only for participants aged 65 years and older (Table 3). The interaction by age group for the association between baseline CIMT and change in global cognition composite score was not statistically significant (*p* = 0.21). No other associations between baseline CIMT and change in cognitive domain composite scores assessed over 2.5 years were observed for either age group. As with the cross-sectional analysis at 2.5 years, trial treatment assignment did not affect estimates of the longitudinal associations between CIMT and cognitive function over 2.5 years.

The average CIMT at 2.5 years across all three clinical trials (0.79 ± 0.12 mm) increased by 0.02 mm from the average CIMT at baseline. There were no significant associations between change in CIMT and change in cognition in models adjusting only for baseline cognitive function scores.

## Discussion

This post hoc longitudinal analysis of three randomized trials revealed a weak inverse association between CIMT at baseline and change in global cognition composite score assessed 2.5 years later. The global cognition composite score includes cognitive tests comprising the executive function, visual memory, and verbal memory composite scores, which may cumulatively strengthen the overall association between CIMT at baseline and change in global cognition composite score from baseline to 2.5 years compared to associations with specific cognitive domains.

When the longitudinal analysis was stratified by age group, the weak inverse association was statistically significant among participants aged 65 years and older. Age was inversely associated with a decline in global cognition at 2.5 years in our data (Pearson’s *r* = − 0.19), and older-aged adults typically exhibit greater levels of cognitive decline compared to their middle-aged counterparts [10]. Increasing age is also known to be associated with higher CIMT [31] and is evident in our data (Pearson’s *r* = 0.37). It is possible that associations between CIMT and cognition become apparent once CIMT reaches a certain thickness, as in the older participants in this sample.

Stronger associations of cognition with CIMT were observed longitudinally over 2.5 years than cross-sectionally at baseline or at 2.5 years. In this population of healthy middle- to older-aged adults, changes in cognition may be a more sensitive indicator of vulnerability to vascular health than a measure of cognition at a single time point. Cross-sectional associations between CIMT and cognitive functions at baseline and at 2.5 years were not observed. In a previous cross-sectional analysis using data from BVAIT, baseline measures showed that thicker CIMT was weakly associated with lower verbal learning abilities, but not with global cognition [5]. Because of clinical trial inclusion/exclusion criteria and the demands of participation, persons with higher levels of cognitive impairment would not have been enrolled despite possibly having cardiovascular disease risk factors. This may have created a healthy selection bias that reduced cross-sectional baseline associations.

Atherosclerosis may adversely affect cognition through several mechanisms, including cerebral hypoperfusion, as blood vessel stenosis may result in reduced blood flow and oxygen supply to the brain. Reduced intracerebral perfusion can damage brain tissue and lead to declines in cognitive function [32]. Indeed, low cerebral blood flow velocity has been found to be associated with cognitive decline [33]. Higher CIMT is a risk factor for subclinical brain infarcts, or silent strokes, which are in turn associated with declines in cognitive function and an increased risk for dementia [34–36]. Silent strokes may not have noticeable symptoms and are thought to be common among older people, with the estimated prevalence ranging from 5 to 62% in populations with mean ages of 54 to 79 years old, respectively [37]. This mechanism is entirely plausible in this study population of otherwise healthy middle- to older-aged adults. Although this study did not directly measure cerebrovascular stenosis through methods such as brain MRI or transcranial Doppler, carotid artery atherosclerosis serves as a surrogate marker for intracerebral atherosclerosis [10]. CIMT used in this study was developed and validated with serial quantitative coronary angiography as a measure of generalized atherosclerosis [38] and CIMT is a predictor of clinical cardiovascular events [39]; however, unilateral measurement of CIMT from plaque-free common carotid artery may underestimate the atherosclerosis burden of cerebrovascular arteries.

## Limitations

Aspects of this study that would limit the generalizability include the inclusion of more women than men due to the convenience of available data from three clinical trials, two of which only enrolled postmenopausal women. Because of the focus on postmenopausal women in the WISH and ELITE studies, the study sample included few individuals who were middle-aged (e.g., 40–49) or elderly (> 80 years of age). In addition, given clinical trial selection criteria, individuals with a history of cardiovascular disease and diabetes were excluded. As such, the findings of this study may not be generalized to individuals with cardiovascular disease, premenopausal women, or elderly individuals > 80 years. Lastly, the findings may not be generalizable to populations outside of the USA.

## Conclusions

In this longitudinal study of 1495 healthy adults with a mean age of 61 years, greater carotid artery intima-media thickness at baseline had a weak, statistically significant inverse association with change in global cognition assessed over 2.5 years. This study provides evidence that subclinical atherosclerosis of the carotid artery may be a modifiable correlate of cognitive decline in middle and older age. Further research is needed to expand and support these findings in more representative samples. Given that the number of people with dementias is expected to increase in the coming decades, early detection and management of cardiovascular disease could reduce the risk for cognitive impairment in older age.

## Data Availability

The datasets used and analyzed during the current study are available from the corresponding author on reasonable request.

## Abbreviations

ApoE: Apolipoprotein E
BMI: Body mass index
BVAIT: B-Vitamin Atherosclerosis Intervention Trial
CES-D: Center for Epidemiological Studies Depression Scale
CIMT: Carotid intima-media thickness
ELITE: Early versus Late Intervention Trial with Estradiol
HDL: High-density lipoprotein
LDL: Low-density lipoprotein
SBP: Systolic blood pressure
USC: University of Southern California
WISH: Women’s Isoflavone Soy Health trial
